# Loss of Microglia and Impaired Brain-Neurotrophic Factor Signaling Pathway in a Comorbid Model of Chronic Pain and Depression

**DOI:** 10.3389/fpsyt.2018.00442

**Published:** 2018-10-04

**Authors:** Cuizhen Zhu, Jinjie Xu, Yezhe Lin, Peijun Ju, Dongxia Duan, Yanjia Luo, Wenhua Ding, Shengnan Huang, Jinghong Chen, Donghong Cui

**Affiliations:** Shanghai Key Laboratory of Psychotic Disorders, Shanghai Mental Health Center, Shanghai Jiao Tong University School of Medicine, Shanghai, China

**Keywords:** microglia, BDNF, comorbidity, chronic pain, depression

## Abstract

Major depressive disorder (MDD) and chronic pain are two complex disorders that often coexist. The underlying basis for this comorbidity is unknown. In the current investigation, microglia and the brain-derived neurotrophic factor (BDNF)-cAMP response element-binding protein (CREB) pathway were investigated. A comorbidity model, with characteristics of both MDD and chronic pain, was developed by the administration of dextran sodium sulfate (DSS) and the induction of chronic unpredictable psychological stress (CUS). Mechanical threshold sensory testing and the visceromotor response (VMR) were employed to measure mechanical allodynia and visceral hypersensitivity, respectively. RT-qPCR and western blotting were used to assess mRNA and protein levels of ionized calcium-binding adaptor molecule 1 (Iba-1), nuclear factor-kappa B (NF-κB), nuclear factor of kappa light polypeptide gene enhancer in B-cells inhibitor, alpha (IκBa), BDNF, and CREB. In comorbid animals, mechanical allodynia and visceral hypersensitivities were significant with increased mRNA and protein levels for NF-κB-p65 and IκBa. Furthermore, the comorbid animals had deceased mRNA and protein levels for Iba-1, BDNF, and CREB as well as a reduced number and density of microglia in the medial prefrontal cortex (mPFC). These results together suggest that DSS and CUS can induce the comorbidities of chronic pain and depression-like behavior. The pathology of this comorbidity involves loss of microglia within the mPFC with subsequent activation of NF-κB-p65 and down-regulation of BDNF/p-CREB signaling.

## Introduction

Major depressive disorder (MDD) and chronic pain are two complex disorders that often coexist. Chronic pain is very prevalent in patients with MDD (1). Further, depression can increase the risk for chronic pain and as well chronic pain can increase the risk for depression (2, 3). Previous studies showed that 9.3–23% of patients with chronic inflammatory diseases, such as gastrointestinal disease and arthritis, also present with depression (4–6). Chronic pain often occurs in chronic inflammatory diseases, especial inflammatory bowel disease (IBD) (7, 8). Evidence suggests that the gut can be likened to a second human brain and that the gut-brain axis is a bidirectional communication system (9). It is worth noting that patients with chronic pain and depression respond poorly to current treatment regimens (10) and that the pathological basis for the comorbidity of MDD and chronic pain is unknown.

Brain functional integrity is dependent upon not only neurons but also on microglia, which are a major immune cell population within the central nervous system (CNS). Microglia play an important role in resistance to infection, chronic stress, and chronic pain conditions (11). Previous studies have demonstrated microglia to support homeostatic neuronal function and to modulate synaptic plasticity, at the level of the individual synapse to the level of neural circuits (12)_._ Persistent exposure to psychological stress has a profound impact on the immune response, which perturbs microglia function and may contribute to MDD (13–15). Many studies have shown acute stressful conditions to directly induce microglial activation. Stress (duration ranging from 4–21 days) induces microglial proliferation, disrupts neuron-microglia interactions, and produces synaptic deficits in stress-responsive brain regions (16, 17)_._ Chronic unpredictable stress (CUS) can prompt an increase in microglia apoptosis, which reduces microglia cell numbers and a microglial dystrophic morphology. Treatment of CUS-exposed mice with antidepressant drugs can stimulate hippocampal microglial proliferation and reverse CUS-induced microglial decline as well as depressive-like behavior (13, 18). These observations suggest that with chronic stress microglia undergo a dynamic change that may induce a depressive-like condition in rodents and that microglia dysfunction may play a role in chronic stress-induced depression.

Inflammatory cytokines, such as interleukin (IL)-6, IL-1β, IL-8, and tumor necrosis factor (TNF)-α as well as the transcription factor NF-κB, function in the pathogenesis and development of chronic pain and depression (19, 20)_._ Further, microglia can act as neurotrophic cells with evidence demonstrating that brain-derived neurotrophic factor (BDNF), hepatocyte growth factor (HGF), and basic fibroblast growth factor (bFGF) were partially responsible for neurotrophic effects on dopaminergic neurons (21). BDNF is secreted constitutively by non-stimulated microglia with secretion of BDNF enhanced by inflammatory factors (22). Further, BDNF mediates nociception by activation of a number of intracellular signaling molecules including Protein Coding Phospholipase C Gamma 1 (PLCg-1), cAMP response element binding protein (CREB), and AKT (23, 24). The relationship of these cellular signaling pathways to the comorbidity of chronic pain and depression, induced by colonic inflammation and psychological stress, is not well understood.

Considering the depression and chronic pain comorbidity in the context of gut inflammation and prolonged psychological stress, it is important to examine the underlying molecular signaling pathway. The aim of this study was to evaluate the BDNF-CREB signaling pathway mediated by microglia in the medial prefrontal cortex (mPFC) as defined after colonic inflammation and psychological stress. The hypothesis was that gut inflammation and prolonged psychological stress would excessively stimulate the innate immune system, resulting in CNS inflammation and increasing pain and negative emotion. In parallel, the symptoms of depression and chronic pain as well as abnormal signal pathway underlying can be normalized by the antidepressant duloxetine, which might be potentially involved in repair mechanisms in the CNS.

## Methods

The experiments were conducted in 6 weeks old male C57/BL6 mice (18–20 g) and purchased from Shanghai Silaike experimental animal limited liability company. All mice were housed with six animals per cage at a constant temperature (22–25°C) and humidity of 25–70% under a light and dark cycle of 12 h. They were given standard food and drinking water *ad libitum*. Animal experiments were performed in accordance with international guidelines and approved by the Experimental Animal Committee of Shanghai Jiao Tong University School of Medicine. All Animals were randomly assigned to naïve, DSS colitis, stress, and comorbidity group (all *n* = 6).

### Drug administration

Duloxetine [(S)-Duloxetine Hydrochloride] (Sigma-Aldrich, St. Louis, MO, USA) was dissolved in a saline solution (0.9%). The comorbidity group was treated with duloxetine at a dose of 10 mg/kg for 10 days, day 28 through 39. This dose was chosen based on the stable antidepressant action of duloxetine reported in previous studies. Control mice group were injected with the same volume of saline (25).

**Figure 1:**
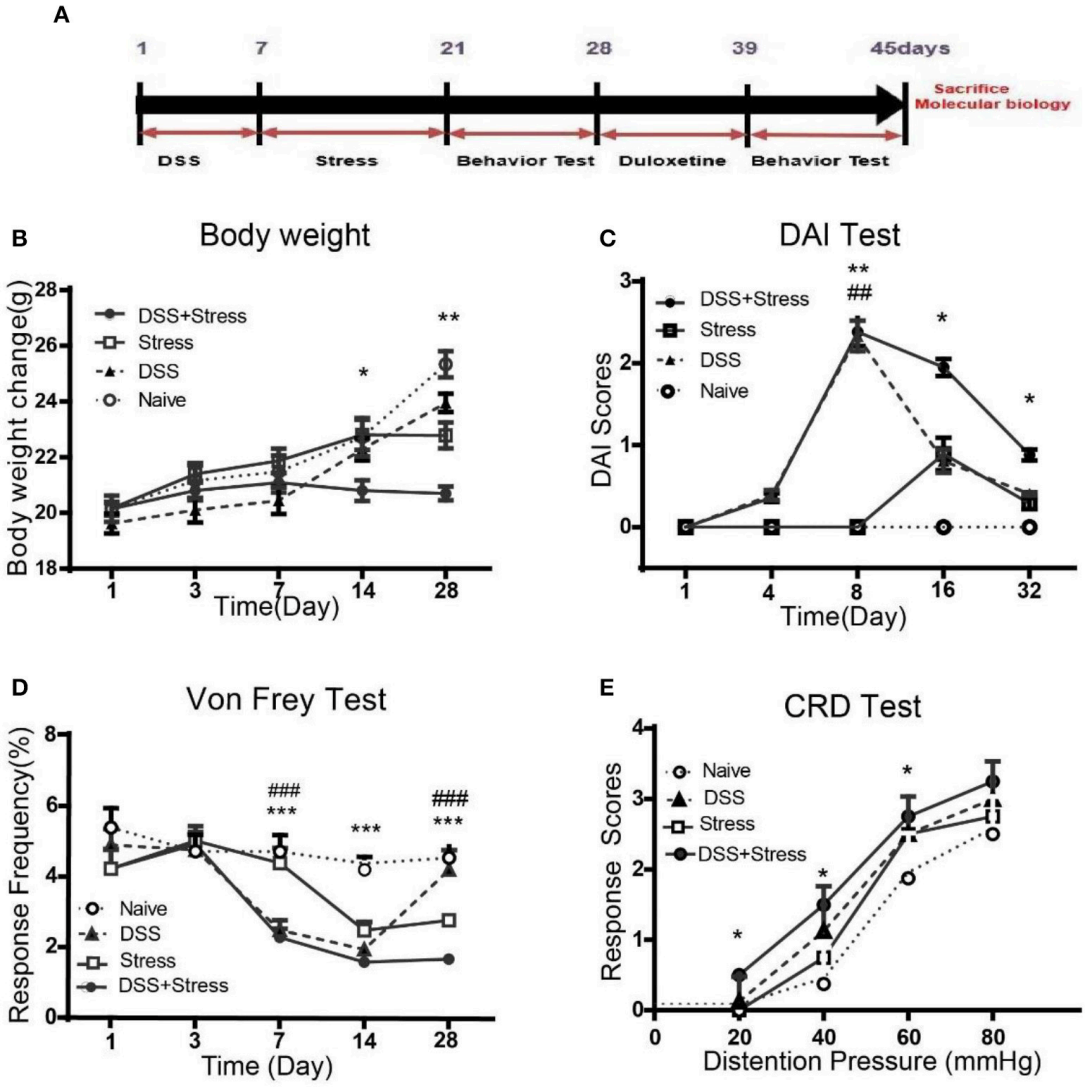
Body weight, DAI scores, mechanical threshold sensory, and visceromotor response in different groups. **(A)**Time of DSS, CUS exposure, behavioral testing, and duloxetine treatment (*n* = 6 per group). **(B)** Body weight was performed on days 1, 3, 7, 14, and 28. **(C)** DIA scores were assessed on days 1, 4, 8, 16, and 32. **(D)** Mechanical allodynia was assessed using nylon VF filaments, tests were performed on days 1, 3, 7, 14, and 28. **(E)** Visceral hypersensitivity was assessed by VMR to 20, 40, 60, and 80 mmHg CRD pressure at the 28th day. Statistical comparisons were performed with one-way ANOVA followed by Dunnett's *t*-test and two-way ANOVA followed by *post hoc* Bonferroni. *p < 0.05, **p < 0.001, ***p < 0.0001, compared with the naïve group;^##^*p* < 0.001, ^###^*p* < 0.0001 compared with the comorbidity group. DAI, Disease activity index; VF, von Frey; CRD, colorectal Distention; VMR, visceromotor response.

Dextran sodium sulfate (DSS; MP Biomedical LLC, USA) is one of the most widely used agents for chemical-induction of colitis in experimental models of IBD. The DSS colitis group and comorbidity group were given oral administration of 5% w/v DSS in drinking water for 5 days to induce colonic inflammation. From day 6 onward all animals received normal drinking water(26) (Figure 1A).

### CUS procedure

The stress group and the comorbidity group were exposed to a variable sequence of five unpredictable psychological stressors, two times per day for 14 days. The stressors included cages tilted at 45 degrees for 2 h, water avoidance stress (WAS; 60 min), forced swimming stress (FSS; 10 min), 4°C cold stress for 45 min, and restraint stress for 4 h, as described in previous studies (27, 28) (Figure 1A).

### Disease activity index (DAI)

DAI was quantified with a clinical score assessing weight loss, stool consistency, and fecal blood (measured by occult blood test paper), no weight loss was counted as 0 points, weight loss of 1 to 5% as 1 point, 5 to 10% as 2 points, 10 to 20% as 3 points, more than 20% as 4 points. For stool consistency 0 points were given for normal, 1 point were given for soft but still formed, 2 points were given for very soft, 3 points were given for diarrhea. Bleeding was scored 0 points for negative occult blood test, 1 point for positive occult blood test, 2 points for blood traces in stool visible, 3 points for rectal bleeding. These scores were added and divided by 3, forming a total clinical score that ranged from 0.0 (healthy) to 4.0 (maximal activity of colitis) (29)_._

### Mechanical threshold sensory test

Mechanical allodynia was assessed using nylon von Frey (VF) filaments (Aesthesio Precision Tactile Sensory Evaluator; DanMic Global LLC). Mice was placed in a Plexiglas box (23 × 18 × 14cm) with 0.8 cm mesh flooring. The “paw withdrawal reflex” was defined as a rapid withdrawal of the paw when VF filaments were touched to the plantar surface of the hind paw. If a response to a given graded fiber was not observed, the next stiffer fiber was applied to the same paw until a fiber evoking response was found. The lowest force leading to at least three withdrawals in five trials was defined as the withdrawal threshold to mechanical stimuli (30). VF tests were performed on day 1, 3, 7,14, and 28.

### Visceromotor response (VMR)

A sphygmomanometer was used to measure VMR, 2 cm balloon was gently inserted into the colon under isoflurane anesthesia with the base of the balloon 5 mm proximal to the anus, mice received a series of 10 sec gastric balloon distensions: 20, 40, 60 and 80 mmHg with 2 min intervals between distensions (31). After the experiments, the balloon and the connecting cable were removed under isoflurane anesthesia, and the animals were returned to their home cage.

### Assessment of depressive-like behaviors sucrose preference test (SPT)

Anhedonia is a core behavioral indicator of clinical depression in normally rewarding stimuli, SPT used to assess symptoms of anhedonia. The first day mice were habituated to water bottles and 1% sucrose solution bottles; then at the second day food and water taken away from the mice. On the third day, the bottles of water and bottles of 1% sucrose solution were given to the mice. The amount of water and 1% sucrose solution consumed was measured at the first 1 h and 24 h, and the bottles were reversed to control for side preference. The mean sucrose preference of the two different time was calculated based on the ratio of sucrose solution consumption to total liquid consumption. The sucrose preference rate = [sucrose consumption/(water consumption +sucrose consumption)]100% (32).

### Forced swimming test (FST)

FST was used to evaluate behavioral despair in one set of animals. Mice were placed individually inside a glass cylinders (25 cm in height, 14 cm in diameter), that contained 20 cm of water maintained at ~23°C ± 2 and were forced to swim for 6 min: 2 min to adapt to the environment and 4 min to record the immobility. Increase of immobility in rodents in the FST has associated to helplessness state (33).

### Tail suspension test (TST)

Rodent animal, an adhesive tape was placed 1 cm away from the tip of the tail, then mice were suspended in the hook of the tail suspension test box and 60 cm above the surface of table. The total duration of the test (6 min) can be divided into periods of agitation and immobility. Mice were considered immobile only when they hung passively and were completely motionless (34).

### Open field test (OFT)

OFT is aim to assess basal locomotor activity of animals, animal was placed individually in a circular arena with transparent walls (diameter: 30 cm; height: 50 cm) during 10 min (35). The total distance traveled and frequency entering the center were recorded and analyzed.

### Quantitative RT-PCR

Total RNAs were extracted from the prefrontal cortex, and then isolated with Trizol reagent. BioRT cDNA First Stand synthesis kit(Bioer technology, Hangzhou, China) was used to synthesize cDNA. RT-PCR was performed using LightCycler® 480 SYBR Green PCR Master Mix (Roche,USA) and analyzed using LightCycler® 480 SYBR Green SoftwareII. The expression of respective genes was normalized to that of glyceraldehyde phosphate dehydrogenase (GAPDH) within the same sample. The primers used were as follows: 5′-CGTGTCTGCACCTAGCCTCTATC-3′ and 5′- GCGAAACCAGGTCAGGATTC-3′ (IκBα), 5′-CAGGACCAGGAACAGTTCGAA-3′ and 5′-CCAGGTTCTGGAAGCTATGGAT-3′ (NF-κB-p65), 5′-TCGGTTGCATGAAGGC-3′ and 5‘- GGTTTTCTTCGTTGGGC-3′ (BDNF), 5′-GAATGATGCTGGGCAAGAGA-3′ and 5′-CAGTTGGCTTCTGGTGTTC-3' (Iba-1), 5′-TACAGGGCCTGCAGACATTAACCA-3′ 5′-ATTCTCTTGCTGCCTCCCTGTTCT-3′ (CREB), 5′-ATGTGTCCGTCGTGGATCTGA-3′ and 5′- ATGCCTGCTTCACCACCTTCT-3′ (GAPDH). RT-PCR was first identified to be specific using the melting curves and the relative expression of each mRNA level was calculated with the 2^−ΔΔ*CT*^ method after normalizing Ct (cycle threshold) values with GAPDH.

### Western blotting analysis

The sample from brain tissues were separated by SDS-PAGE and transferred to PVDF filter (0.45 μm, millipore, USA). The membrane was incubated overnight at 4°C with primary antibodies including T-NF-κBp65/p-NF-κBp65(Ser536)/T-IκBa/p-IκBa/T-CREB/ p-CREB/ GAPDH (1:1000, Cell signaling Technology, USA), BDNF/Iba-1 (1:500, Abcam' RabMAb® technology), followed by incubation with appropriate HRP-conjugated secondary antibodies and finally visualized by chemiluminescence (ECL Advance; Amersham Biosciences), and exposure to X-ray films for 10 s −5 min.

### Immunohistochemistry

Mice were anesthetized with chloral hydrate in 10% and perfused with 0.9% saline solution followed by 4% paraformaldehyde (PFA) solution (Sheng Gong, China) in 0.01 M phosphate buffer (PBS). The collected tissue was post-fixed overnight at 4°C in 4% PFA and dehydrated by gradient sucrose in PBS and then cryoprotected at 4°C in 30% sucrose in PBS. 20-μm-thick coronally sections of the prefrontal cortex were cut and pasted on glass slides, after blocking solution (0.3% Triton X-100, 3% donkey serum in PBS) for 1 h at room temperature, the slices were incubated with primary antibodies: rabbit anti-Iba1 (1:500; Wako, Japan) in solution (0.3% Triton X-100, 3% donkey serum in PBS) at room temperature, overnight. After washing with PBS for 3 × 5 min, the slices were exposed to the secondary antibody solution containing goat anti-rabbit Alexa Fluor 488/549 (1:500, Invitrogen, Carlsbad, CA, USA) for 2 h at room temperature, and 4′,6-diamidino-2-phenylindole (DAPI, 1: 1,000; Sigma, St. Louis, MO, USA) for 15 min at room temperature. A confocal microscope (Leica, TCS SP2, Germany) was used to capture the fluorescent images. Immunofluorescence intensity was calculated by Image J (Wayne Rasband, National Institute of Health, USA).

### Statistics

Data from behavioral and disease activity indices are presented as mean ± standard deviation (SD). The entire behavioral test statistical analysis of data was carried out by one-way analysis of variance (ANOVA), followed by *post hoc* Tukey's Multiple Comparison. Von Frey test and colorectal distension pressure (CRD) test were analyzed by two-way ANOVA with *post hoc* Bonferroni mean comparisons. Differences were considered statistically significant if the *p* < 0.05. The statistical program used was Graph Pad Prism 6.0 Version for Windows, Graph Pad Software (San Diego, CA, USA).

## Results

### Weight loss and hyperalgesia of the comorbidity group were more severe than other groups

As previously described, the symptom of colitis, such as body weight loss, stool consistency, and severity of rectal bleeding, were scored from zero to 4 to determine the DAI. Weight loss is an independent risk factor for future development of comorbidity of depression and chronic pain. Results showed that the body weight loss was most severely decreased after DSS intake in the comorbidity group, even at the 28 days, the body weight of the comorbid mice was still lower than other three groups [*F*_(12, 100)_ = 4.14, *p* < 0.001; Figure 1B]. At 8 days, DAI was greater for DSS-treated mice than controls [*F*_(12, 100)_ = 4.16, *p* < 0.001; Figure 1C]. Moreover, the DAI score for the comorbidity group was still significantly higher than other groups at 32 days [*F*_(3, 12)_ = 8.164, *p* < 0.05; Figure 1C].

At 7 days post DSS-treatment, the DSS and the comorbidity group showed a significant increase in mechanical pain sensitivity, exhibiting a reduction in the mechanical threshold sensory response when compared to the naïve and stress groups [*F*_(12, 100)_ = 4.10, *p* < 0.0001; Figure 1D] in the contextual paradigm of the VF test. Additionally, *post hoc* Turkey's Multiple Comparison Test analysis indicated that mechanical pain sensitivity of the DSS and the comorbidity group was higher compared to the stress and the naïve groups (*p* < 0.0001; Figure 1D) at 14 days. However, at 28 days, mechanical pain sensitivity results revealed no significant changes between the DSS and the naïve groups, while the comorbidity group was higher than the other groups (*p* < 0.0001; Figure 1D). Visceral hypersensitivity was assessed by VMR to 20, 40, 60, and 80 mmHg colorectal distension pressure (CRD) at 28th day, compared with the other groups, the comorbidity group exhibited higher [*F*_(1.836, 5.509)_ = 19.78, *p* < 0.05; Figure 1E] VMR with 20, 40, 60 mmHg CRD pressures. These data suggest that the visceral hypersensitivity was highest in the comorbidity group.

### Depression-like behavioral phenotypes of the comorbidity group were more severe than other groups

**Figure 2:**
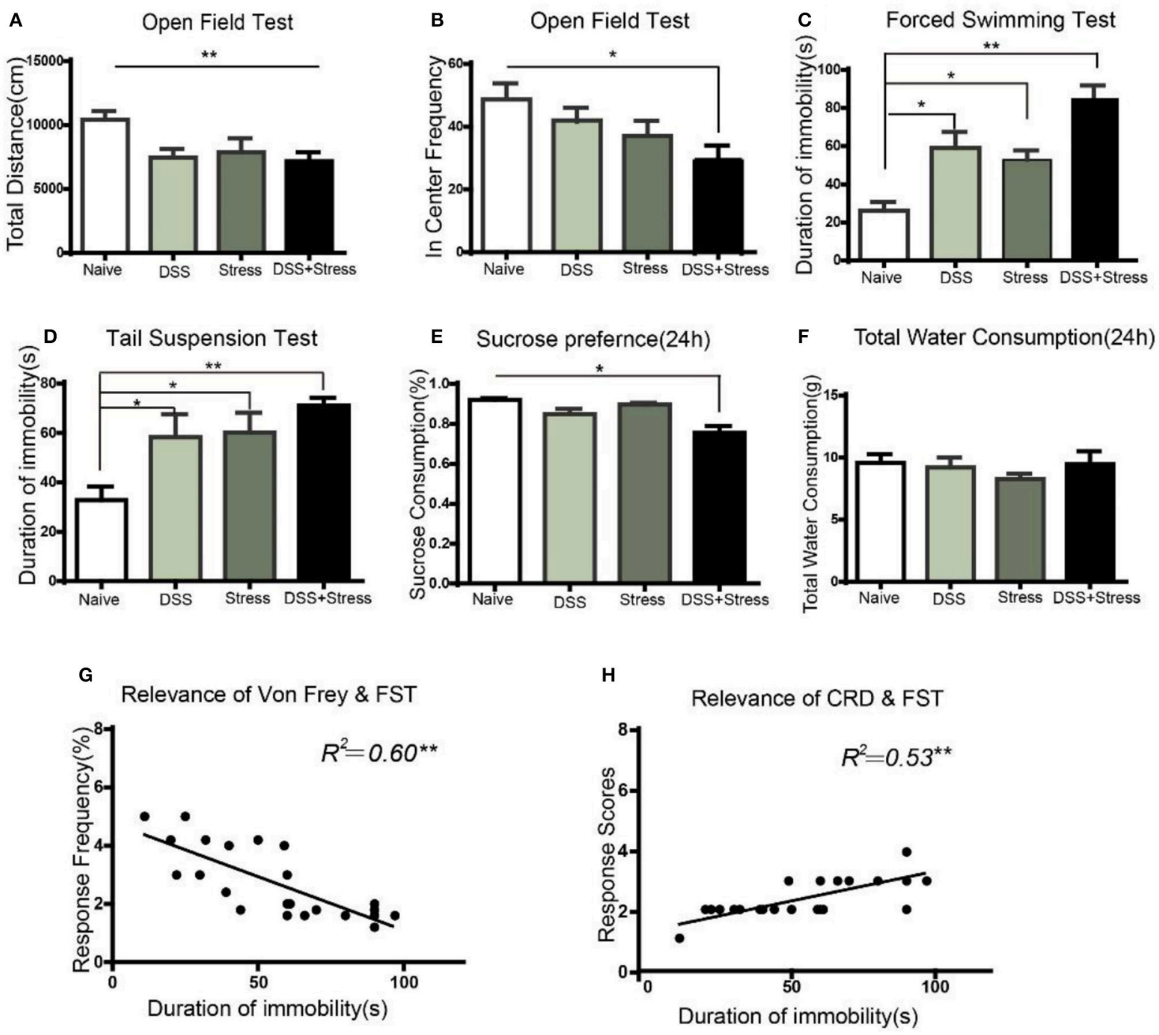
Depression-like behavior in different groups. **(A)** The total distance of movement reduced in the DSS, stress, and comorbidity groups in OFT. **(B)** In center frequency reduction in the comorbidity group in OFT. **(C,D)** Increased immobility time in the FST and TST in DSS, stress, and comorbidity groups. **(E)** Decreased sucrose preference in 24 h in the comorbidity group. **(F)** No difference on total fluid consumption in 24 h. **(G,H)** Correlation analysis of mechanical allodynia and visceral hypersensitivity with immobility time in FST. Statistical comparisons were performed with one-way ANOVA followed by Dunnett's *t*-test and two-way ANOVA followed by *post hoc* Bonferroni. *p < 0.05, **p < 0.001, compared with the naïve group; OFT, open field test; FST, forced swim test; TST, tail suspension test; SPT, sucrose preference test.

In order to study the depression-like behaviors of mice subjected to DSS and CUS, we performed series of behavioral tests. OFT was used to evaluate the locomotor activity of the animals. Results showed that total distance decreased in the comorbidity group in comparison to the naïve group [*F*_(3, 20)_ = 7.629, *p* < 0.001; Figure 2A]. *Post hoc* Tukey's Multiple Comparison Test analysis further confirmed that frequency of the comorbidity group into the center area decreased when compared to the naïve group (*p* < 0.05; Figure 2B).

To further assess whether existed increasing helplessness behavior in comorbidity group, we performed FST and TST behavioral tests, which were indicative of depressive phenotypes. In the FST, the comorbidity group exhibited a significant increase in immobility time compared to the naïve group [*F*_(3, 20)_ = 15.66, *p* < 0.001; Figure 2C]. *Post hoc* Tukey's Multiple Comparison Test analysis indicated that the stress and the DSS group showed increased immobility time in comparison to the naïve group (*p* < 0.05; Figure 2C), as well as the comorbidity group showed increased immobility time when compared to the stress and the DSS groups (*p* < 0.05; Figure 2C). In the TST, compared to the naïve group, the comorbidity group showed a significant increase in immobility time [*F*_(3, 20)_ = 5.740, *p* < 0.001; Figure 2D]. *Post hoc* Tukey's Multiple Comparison Test analysis indicated that the DSS and the stress group showed a significantly increased immobility time compared to the naïve group(*p* < 0.05; Figure 2D).

SPT was used to assess the core anhedonia symptoms of depression, and results revealed a significant sucrose preference rate reduction in the comorbidity group compared to the three other groups [*F*_(3, 26)_ = 5.17, *p* < 0.05; Figure 2E] after24 h. *Post hoc* Tukey's Multiple Comparison Test analysis indicated that the comorbidity group showed a significant reduction in sucrose consumption compared to the naïve, stress, and DSS groups (*p* < 0.05; Figure 2E) after 24 h. Nonetheless, the total amount of water consumption were not statistically significant among four groups[*F*_(3, 26)_ = 0.558, *p* > 0.05; Figure 2F]after 24 h.

Taken together, these results suggested that the severity of pain and the depression-like behavioral phenotype of the comorbidity group were more severe than the other groups. Moreover, the multiple linear regression was used to model the relationship between depressive behavior and chronic pain. Our data demonstrated that mechanical pain and visceral hypersensitivity were correlated with the degree of depression(*r* = 0.60, *p* < 0.001; Figure 2G; *r* = 0.53, *p* < 0.001; Figure 2H), suggesting that this model can well imitate the phenotype of comorbidity of depression and chronic pain.

### NF-κBp65 /IκBa were up-regulated and loss of microglia associated BDNF-CREB pathway was down-regulated in the comorbidity group

**Figure 3:**
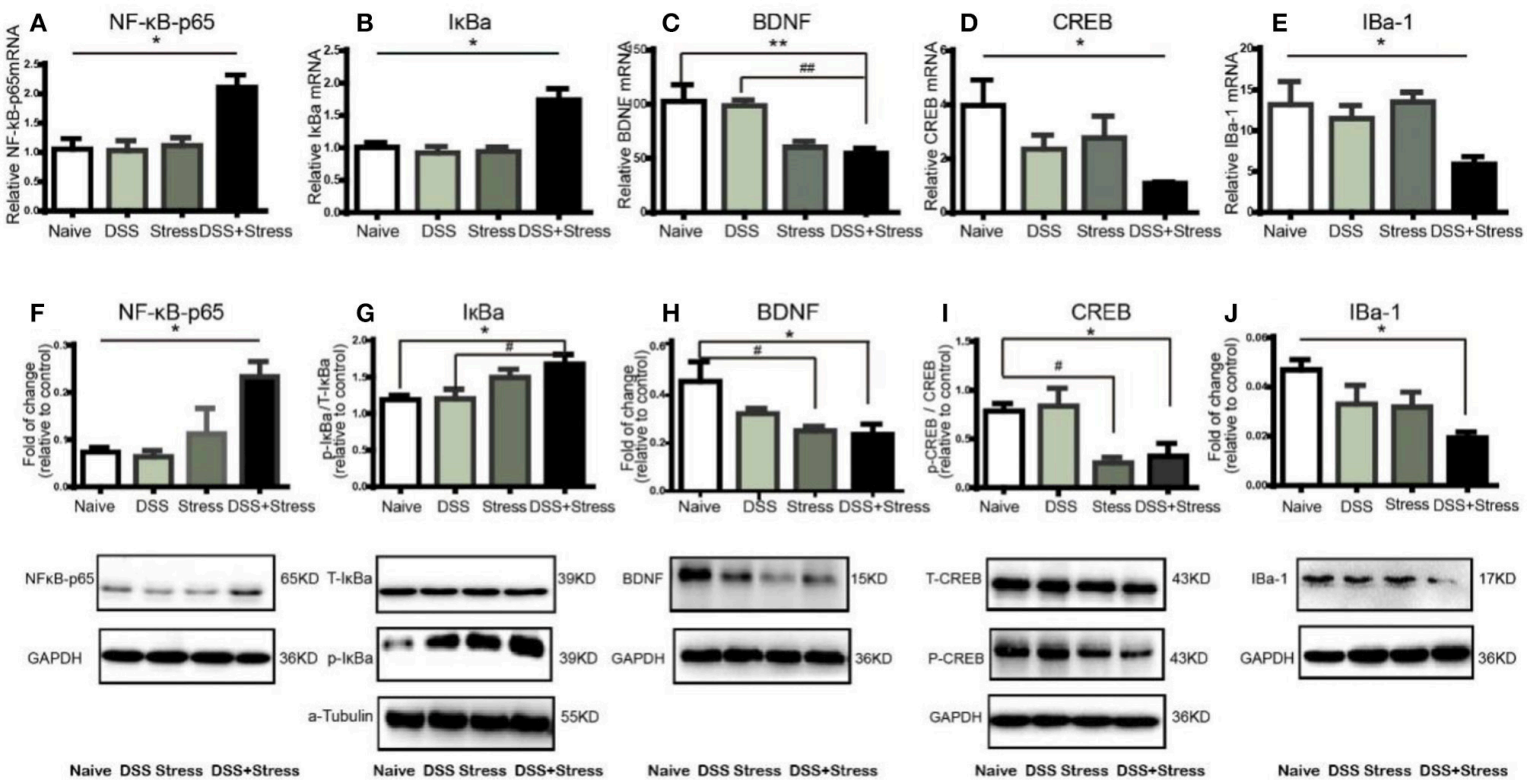
NF-κB, IκBα, BDNF, CREB, and IBA-1mRNA and protein expression in mPFC of mice (*n* = 4 per group). Gene expression was revealed by real-time quantitative polymerase chain reaction as fold change. **(A,B)**NF-κB, IκBα mRNA expression increased in the comorbidity group.**(C–E)** BDNF, CREB, and IBa-1 mRNA expression decreased in the comorbidity group. **(F,G)** Representative western blots of fold changes in NF-κB-p65, T-IκBα, and p-IκBα in the mPFC relative to control. **(H–J)** Representative western blots of fold changes in BDNF, CREB, p-CREB, and Iba-1in the mPFC relative to control. Statistical comparisons were performed with one-way ANOVA followed by *post hoc* Tukey's Multiple Comparison. *p < 0.05, **p < 0.001, compared with the naïve group. ^#^*p* < 0.05,^##^*p* < 0.001, compared with the comorbidity group. NF-κB, nuclear factor-kappa B; BDNF, brain-derived neurotrophic factor; CREB, cyclic AMP response element-binding protein; Iba-1, ionized calcium binding adaptor molecule 1; mPFC, medial prefrontal cortex.

Previous studies showed that several inflammatory mediators can activate the NF-κB signaling pathway during stress and injury. Herein, NF-κBp65 and IκBα mRNA expression increased in the mPFC of the comorbidity group [*F*_(3, 12)_ = 8.55, *p* < 0.05; Figure 3A; *F*_(3, 12)_ = 12.5, *p* < 0.05; Figure 3B]. Consistently, western blotting results showed that the levels of NF-κB-p65 and p-IκBα increased in the mPFC of the comorbidity group, when compared to the other experimental groups [*F*_(3, 10)_ = 7.33, *p* < 0.05; Figure 3F; *F*_(3, 10)_ = 4.392, *p* < 0.05; Figure 3G]. BDNF is one of the most abundant neurotrophic factors in the brain, it has been reported that the proinflammatory cytokines in the brain are considered to lead to BDNF downregulation and subsequent neuronal loss. Interestingly, we found not only the decrease of BDNF mRNA and protein in the mPFC of the comorbidity group [*F*_(3, 11)_ = 10.78, *p* < 0.001; Figure 3C; *F*_(3, 9)_ = 4.959, *p* < 0.05; Figure 3H] but also the decrease of mRNA and protein expression in downstream CREB[*F*_(3, 11)_ = 5.78, *p* < 0.05; Figure 3D; *F*_(3, 9)_ = 5.71, *p* < 0.05; Figure 3I]. In addition, previous studies showed BDNF is secreted constitutively from microglia, we have therefore investigated the changes of microglia cell. Results indicated the mRNA and protein levels of IBa-1 in mPFC of the comorbidity group were dramatically decreased [*F*_(3, 11)_ = 4.941, *p* < 0.05; Figure 3E; *F*_(3, 12)_ = 4.306, *p* < 0.05; Figure 3J]. Taken together, these data suggest that the both NF-κB signaling pathway and the loss of microglia associated BDNF-CREB signaling pathway play an important role in the comorbidity of depression and chronic pain.

### Duloxetine reversal of hyperalgesia, depression-like behavioral phenotypes and abnormal molecular expression of the comorbidity group

**Figure 4:**
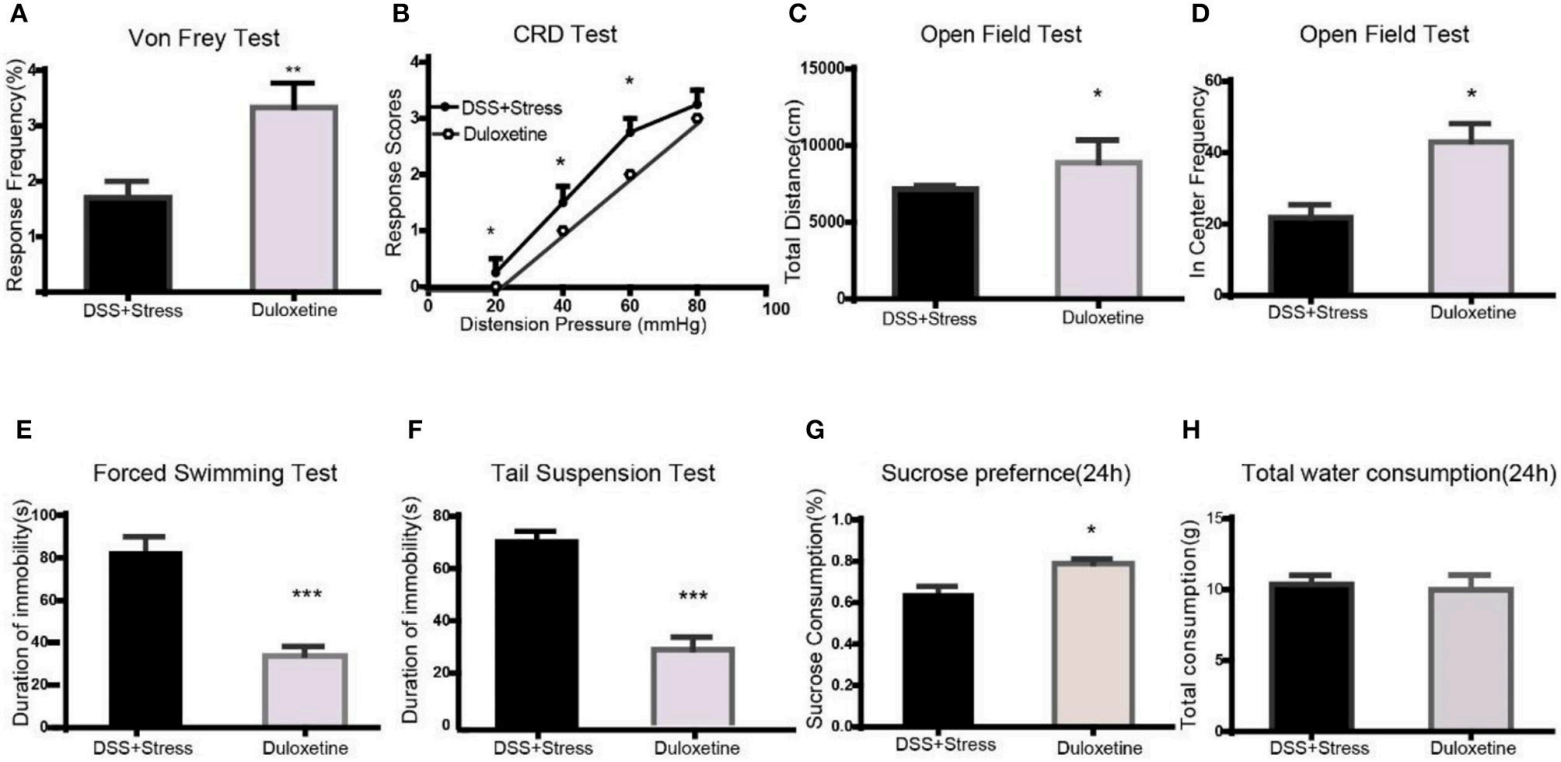
Duloxetine reversed the symptoms of mechanical pain, visceral pain, and depression-like behavior in the comorbidity group (*n* = 6 per group). **(A)** Duloxetine improved mechanical pain sensitivity of the comorbidity group. **(B)** Duloxetine improved visceral pain sensitivity of the comorbidity group. **(C,D)** Duloxetine increased the distance of movement and enter in center frequency in the comorbidity group. **(E,F)** Duloxetine decreased the immobility in the FST and TST. **(G)** Duloxetine increased the sucrose preference in 24 h in the comorbidity group. **(H)** No difference on total fluid consumption in 24 h. Statistical comparisons were performed by *t*-test, mean ± SEM,*p < 0.05, **p < 0.001, ***p < 0.0001 compared with the comorbidity group. FST, forced swim test; TST, tail suspension test.

**Figure 5:**
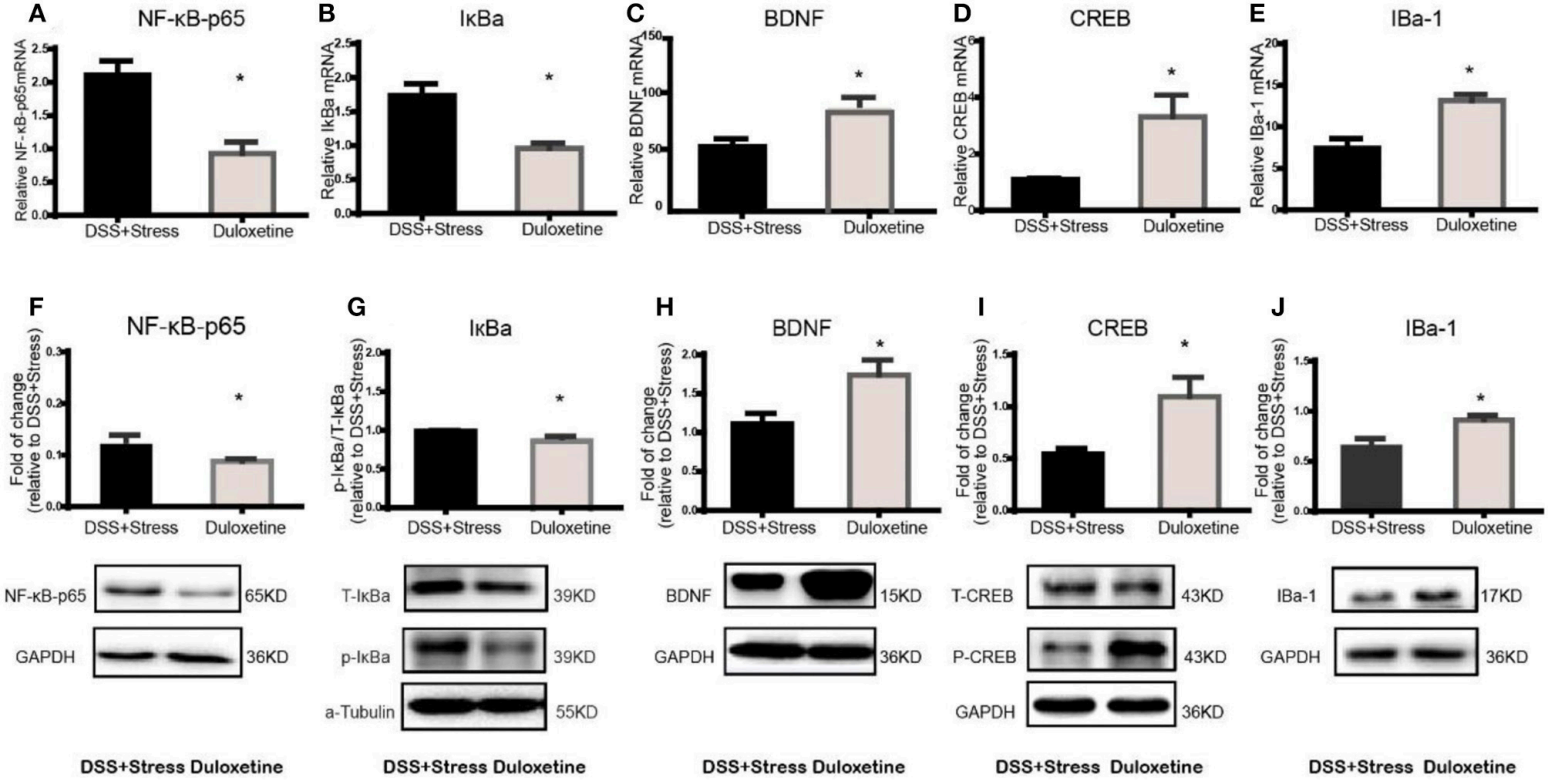
Duloxetine reversed the NF-κB, IκBα, BDNF, CREB, Iba-1mRNA and protein expression in the comorbidity group (*n* = 4 per group). Gene expression was revealed by real-time quantitative polymerase chain reaction as fold change. (**A,B**) Duloxetine can reverse the increased mRNA expression of NF-κB and IκBα in the comorbidity group. (**C–E**) Duloxetine can reverse the decreased mRNA expression of BDNF, CREB and Iba-1inthe comorbidity group. **(F,G)** Duloxetine can reverse the increased protein expression of NF-κB and IκBα in the comorbidity group. **(H–J)** Duloxetine can reverse the decreased protein expression of BDNF, CREB and Iba-1inthe comorbidity group. Statistical comparisons were performed by *t*-test, mean ± SEM, *p < 0.05, compared with the comorbidity group. NF-κB, nuclear factor-kappa B; BDNF, brain-derived neurotrophic factor; CREB, cyclic AMP response element-binding protein; Iba-1, ionized calcium binding adaptor molecule 1; mPFC, medial prefrontal cortex.

Many clinical trials have shown the selective noradrenergic and serotonergic uptake inhibitor, duloxetine, not only attenuates depression-like behavior but also the symptoms of chronic pain. Herein, duloxetine significantly reversed mechanical hypersensitivity visceral hypersensitivity and depression-like behavior in the comorbidity group (Figures 4A-H, Supplementary data 1). Furthermore, duloxetine reversed abnormal molecular expression of the comorbidity group (Figures 5A-J, Supplementary data 2). These data suggested that the loss of microglia and the BDNF-CREB signaling pathway influence the comorbidity of chronic pain and depression induced by colonic inflammation and CUS.

### Immunohistochemistry results of Iba-1 in different groups

**Figure 6:**
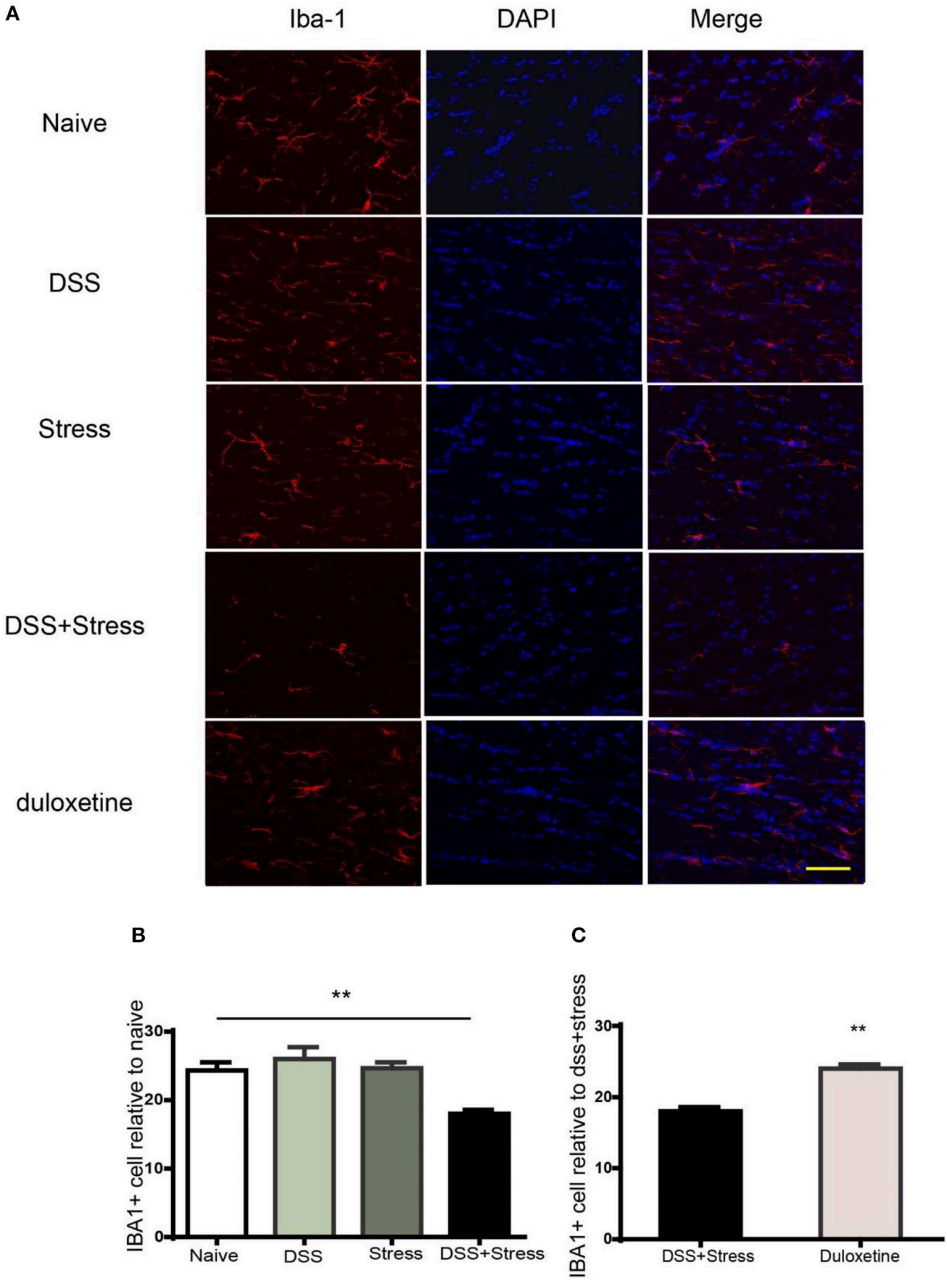
Immunofluorescence staining to detect number of Iba-1 positive cells in the mPFC. **(A)** Representative images of Iba1 (red) in the mPFC (Scale = 40 μm). **(B)** Cell number and fluorescence intensity of Iba1 is decreased in the comorbidity group compared to other groups. **(C)** Duloxetine significantly improved the cell number and fluorescence intensity of Iba-1 in the comorbidity group compared to other groups. The data are expressed as the mean ± SEM of four mice in each group. **p < 0.001 compared to the naïve group. DAPI staining is shown in blue. Iba-1, ionized calcium binding adaptor molecule 1; mPFC, medial prefrontal cortex. DAPI, 4′,6-diamidino-2-phenyl.

The microglia are the most susceptible sensors of brain pathology. In our study, we use the immunohistochemical method to label microglia by Iba-1 (Figure 6A) for assessing microglia in comorbidity. Iba-1 protein is distributed in the cytoplasm of microglia which has the advantage of the wide inter-species stability of its antigenic epitopes. In the immunohistochemistry experiment, we confirmed that the number and density of microglia in the comorbidity group were significantly reduced when compared to the other groups (Figure 6B). Furthermore, duloxetine can improve this phenomenon (Figure 6C, Supplementary data 3), these data suggest that the loss of microglia play an important role in the comorbidity of depression and chronic pain.

## Discussion

Clinical studies have shown that patients with MDD are prone to chronic pain and that patients with chronic pain are prone to MDD (36). Chronic pain is considered a predictive factor for MDD (37) with depression-pain symptoms an increasing clinical concern. Several animal models had been developed to mimic MDD or chronic pain but no effective comorbidity animal model for both MDD and chronic pain exists. In our previous studies, we found that DSS not only exacerbated the spontaneous activity of colon-projecting afferent neurons that induce visceral hypersensitivity, but also induced depression-like behaviors after 10 to 20 days of inflammation (38). As such this animal model may contribute to a more complete pathophysiological understanding of the comorbidity of MDD and chronic pain.

In the present study, we found DSS group and comorbid group exhibited pronounced weight loss and symptoms of fluctuating diarrhea, bloody feces, and pain hypersensitivity during the DSS administration. In CUS alone group, CUS also induced significant weight loss and pain hypersensitivity. Nonetheless, the body weight loss gradually recovered in CUS group, DSS group after 28 days, and the results of occult blood feces test were negative. However, in the comorbidity group, weight loss and hyperalgesia improved significantly slower than in the other groups, even on the 32nd day of the experiment, the weight of the comorbid group was still significantly lower than that of the other groups. These results indicated that the duration of somatic discomfort significantly longer than the other groups. Nevertheless, in the later stage when behavioral and molecular tests were performed, the weight loss of mice in comorbid group also restored and bloody feces gets negative, suggesting that body weight loss and anemia might have loose correlation with the observed changes.

To mimic the anhedonia and helpless depression-like behavior, we carried out a fourteen day of consecutive stress (including water avoidance, WAS). As confirmed in our previous studies, WAS has been used to investigate visceral pain (39). When compared to the other groups, severe despair and anhedonia behaviors were found in the comorbidity group. More importantly, the symptoms of depression and chronic pain were strongly associated in the comorbidity group. These results suggest that chronic stress may increase psychological and physiological damage in patients suffering from chronic pain. Furthermore, duloxetine was used to determine whether the symptoms of depression and chronic pain (or symptoms of hyperalgesia and depression-like behavior) could be improved in the comorbidity group. Consistent with numerous clinical studies, depressive and chronic somatic symptoms were relieved after treatment with duloxetine (40, 41). These results demonstrated this comorbidity model of chronic pain and depression could be a useful and valid model.

Microglial function within the CNS has been described (42, 43). Central nerve injury can induce a change in microglia numbers and morphology that are characterized as either M1 or M2 by upregulating the expression of surface proteins such as CD11, CD68, and CD86 (44–47). Microglia release pro-inflammation mediators, such as IL-6, IL-1β, TNF-a, and nitric oxide that are considered links between chronic pain and depression. Further, microglial proliferation often occurs early in disease. During the initial 2–3 days of stress the number and activation status of microglia, in depressive-like mice, undergo dynamic change. However, after 5 weeks of CUS exposure, microglia undergo apoptosis, which reduces their numbers within the hippocampus(13, 18). Several models of persistent neuropathic pain have demonstrated up-regulation of the number and density of microglia, possibly due to migration and proliferation (43, 48). Little is known about microglial variations during comorbid chronic pain and depression. Herein, decreased Iba-1 mRNA and protein expression were found in the mPFC of the comorbid group, which were not found in the other three groups. Furthermore, analysis of Iba-1-labeled microglial fluorescence demonstrated a reduction in microglial numbers and density within the mPFC of the comorbid group when compared to the other groups. Administration of duloxetine to the comorbid group, partially increased mPFC microglia numbers, although microglial processes were shorter than in the control group. Based on these findings, microglial decline and dysfunction occurred at a late stage of CUS, which may provide an explanation for the interaction of persistent physical and psychological stress associated with the comorbidity of chronic pain and depression.

The NF-κB pathway is well known for its role in inflammation, the regulation of cell differentiation, and apoptosis. In the nervous system, the NF-κB-p65/p50 dimer is a prime inducer of pro-inflammatory genes (49). Sustained activation of NF-κB induces a cycle of microglial activation, that can result in brain parenchymal and neuronal damage. Inhibition of microglia inflammation by reduction of NF-κB activation and by the attenuation of TNF-α, IL-1β,IL-6, and reactive oxygen species (ROS) protects the brain from intracerebral hemorrhage and MDD (50, 51). In our present study RT-qPCR and western blotting analysis demonstrated that NF-κB mRNA and protein levels were increased in the comorbidity group. No difference was observed in the other three groups. Notably, previous studies have shown that phosphorylation of IκBa is responsible for its dissociation from NF-κB, after which free IκBa is rapidly degraded (49). Herein, RT-qPCR and western blotting analysis found that IκBa mRNA and protein levels increased in the comorbidity group, suggesting the possibility that additional cellular events serve to activate NF-κB (52). Clinical results have demonstrated duloxetine to regulate the NF-κB pathway in chronic pain-depression patients (53). Hence, the NF-κB pathway could be involved in the comorbidity of chronic pain and MDD induced by chronic stress and colitis.

Many chronic pain and depression studies have begun to assess roles for NF-κB, CREB, and BDNF in central sensitization. Recent findings indicated that NF-κB does not directly interact with CREB but synergistically recruits CREB-binding protein (CBP) which is essential for transcriptional activation. CBP serves as a scaffold between NF-κB and CREB that stimulates transcription of NF-κB, increasing transcription of multiple genes (54). Moreover, C/EBP and CBP can regulate the response of M1 and M2 microglial, respectively. Many studies have demonstrated that peripheral and central nerve injury can induce activation of microglia. Furthermore, activated microglia can upregulate the P2x4 receptor, which activates the p38-mitogen-activated protein kinase (MAPK), leading to the synthesis and exocytotic release of BDNF from microglia (55, 56). More importantly, BDNF is a mediator of synaptic plasticity, is released by nociceptive neurons, and may contribute to the induction of pathological central sensitization. Herein, mRNA and protein levels for BDNF and CREB were reduced in the mPFC of the comorbid and stress groups, consistent with previous studies that showed mRNA and protein expression of BDNF, CREB and their receptors to be reduced in MDD (57). Hence, duloxetine treatment may upregulate the mRNA and protein expression of BDNF and CREB in the comorbidity group. Taken together, gut inflammation and prolonged psychological stress could affect microglia numbers and BDNF- CREB signaling, resulting in pain and negative affect.

## Conclusions

In conclusion, this investigation demonstrated administration of DSS and CUS to produce a comorbid mouse model of chronic pain and depression-like behavior. This comorbid mouse model exhibited a loss of microglia in the mPFC with subsequent activation of NF-κB-p65 and downregulation of BDNF/p-CREB signaling. Analysis of this complex model may lead to an understanding of the pathological basis for these refractory diseases, may potentially identify beneficial treatments, and possibly shed new light on depression–pain associated pathological mechanisms.

## Author contributions

CZ: conception or design of the work, data collection and data analysis and interpretation. JX, YeL, PJ, DD, and YaL: molecular experiment. WD and SH: animal behavior experiment. JC and DC: conception or design of the work, Critical revision of the article and Final approval of the version to be published.

### Conflict of interest statement

The authors declare that the research was conducted in the absence of any commercial or financial relationships that could be construed as a potential conflict of interest.
