# Supplementary figures and images for: Loss of Microglia and Impaired Brain-Neurotrophic Factor Signaling Pathway in a Comorbid Model of Chronic Pain and Depression
Source: Front Psychiatry. 2018 Oct 4;9:442. doi: 10.3389/fpsyt.2018.00442 (PMC6190863; doi:10.3389/fpsyt.2018.00442)

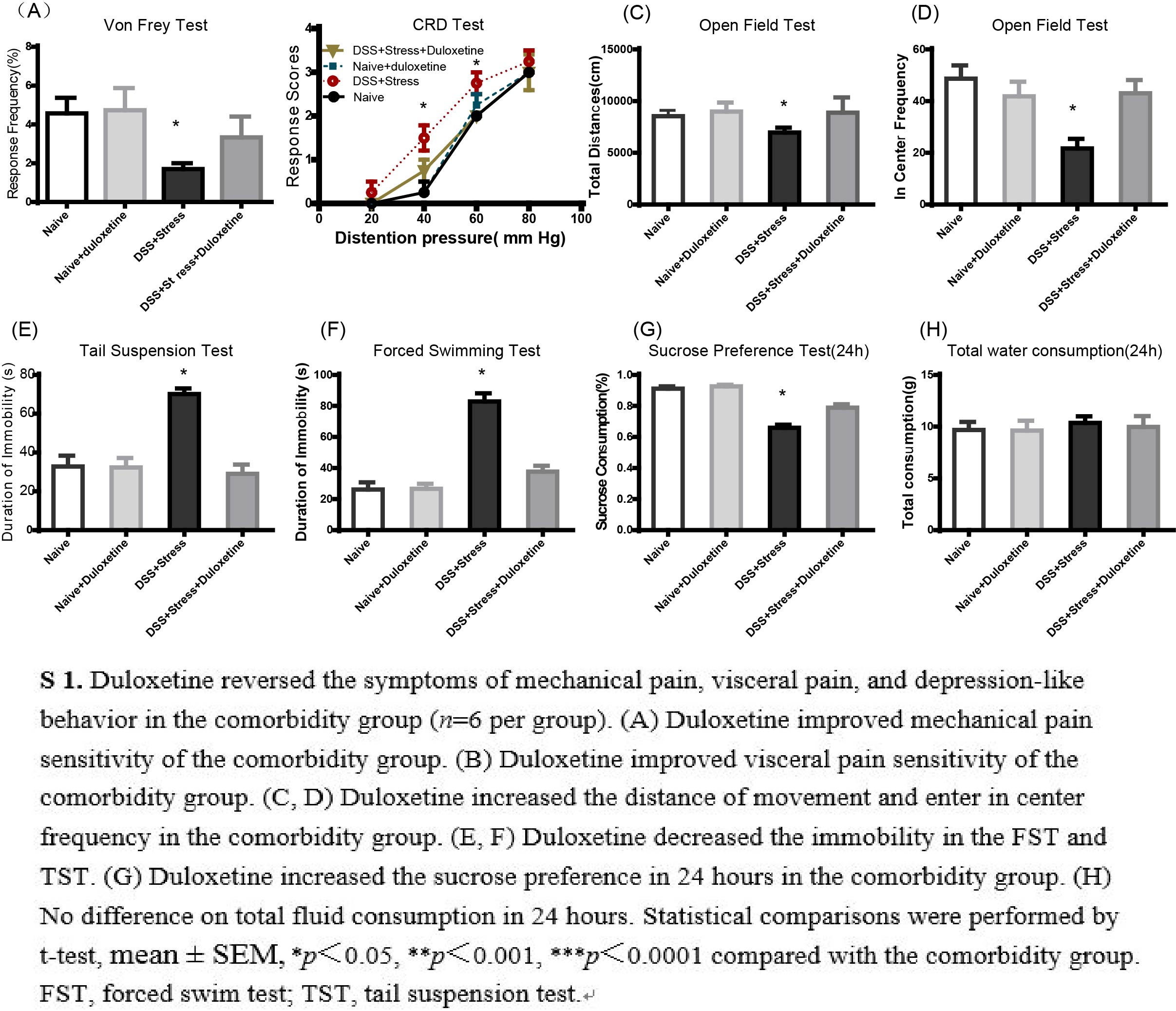

Supplement: Supplementary file 1 [file Image_1.JPEG]

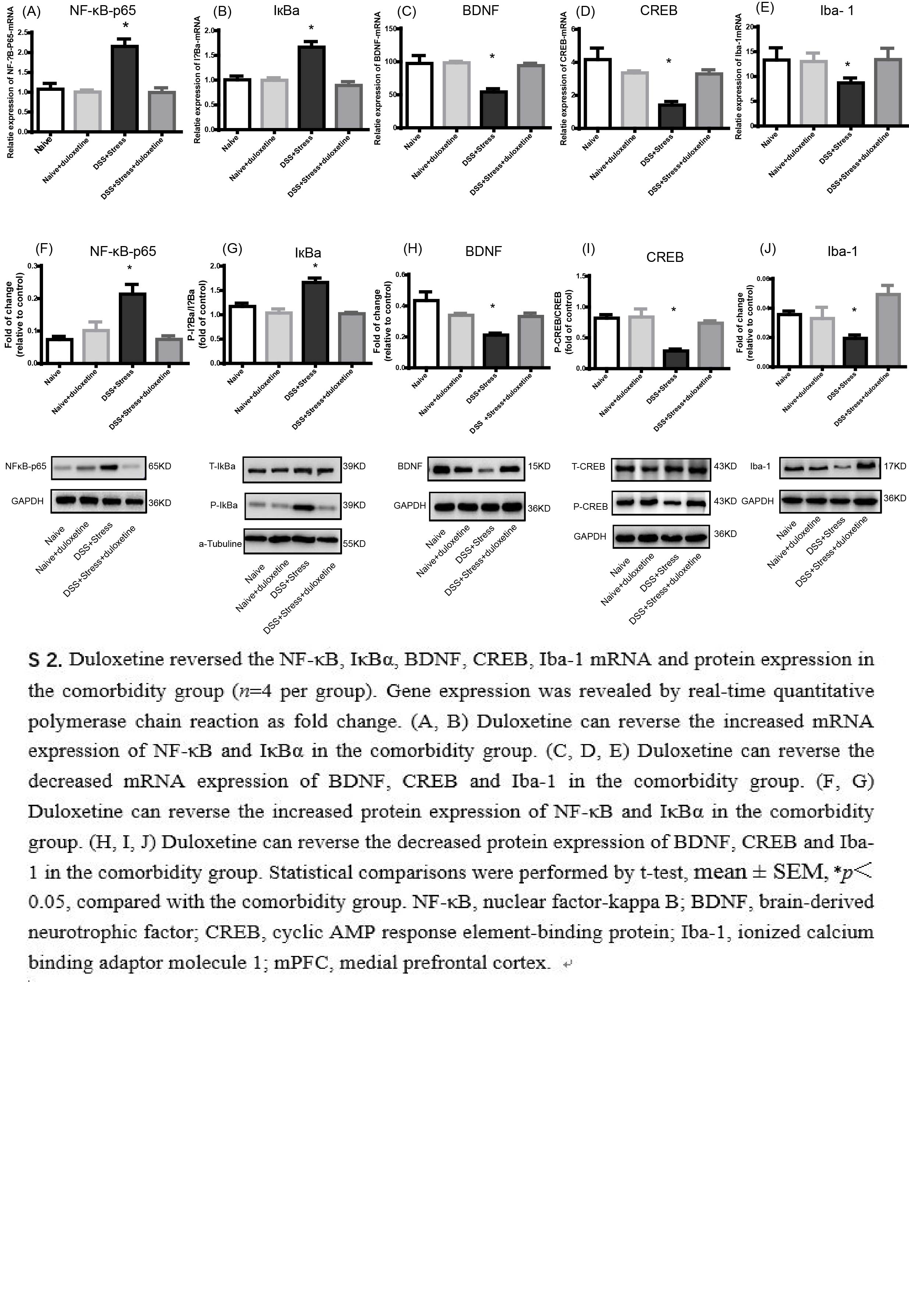

Supplement: Supplementary file 2 [file Image_2.JPEG]

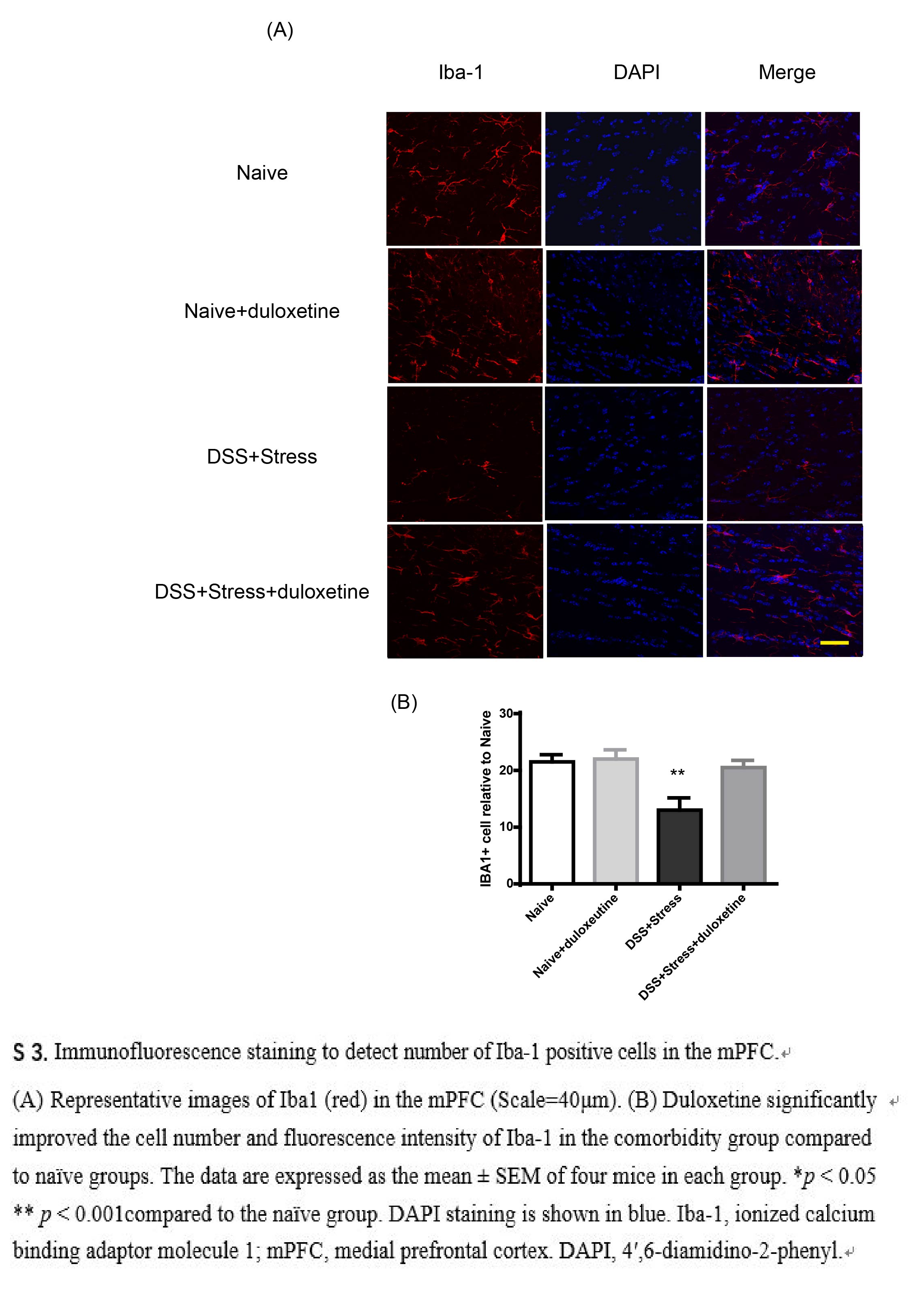

Supplement: Supplementary file 3 [file Image_3.JPEG]
